# A systematic review of the causes and consequences of spreading depolarization in neuroinflammation; implications for neurovascular disorders

**DOI:** 10.1186/s12974-025-03503-6

**Published:** 2025-07-09

**Authors:** Faheem Anwar, Olivia Grech, Caroline W. Mugo, James A. Roberts, Jessica C. Hubbard, Chloe N. Thomas, Alexandra J. Sinclair, Lisa J. Hill

**Affiliations:** 1 Metabolism and Systems Science, School of Medical Science, College of Medicine and Health, University of Birmingham, Birmingham, B15 2TT UK; 2 Department of Biomedical Sciences, School of Infection, Inflammation and Immunology, College of Medicine and Health, University of Birmingham, Birmingham, B15 2TT UK; 3 Department of Neurology, Queen Elizabeth Hospital, University Hospitals Birmingham NHS Trust, Birmingham, B15 2GW UK; 4 Birmingham Biomedical Research Centre, National Institute for Health and Care Research, University Hospitals Birmingham, Birmingham, B15 2TH UK

**Keywords:** Spreading depolarization, Spreading depression, Inflammation, Neuroinflammation, Migraine, Neurovascular disorders, Peri-infarct depolarization

## Abstract

**Background:**

Spreading depolarization (SD) is a wave of neuronal and glial depolarization observed in various neurological conditions, including stroke, traumatic brain injury, subarachnoid haemorrhage, and migraine aura. This depolarization disrupts ion homeostasis, creating high energy demand for recovery. While healthy tissue can compensate, pathological tissue may develop ischemia, worsening brain injury and outcomes. Identifying inflammatory mediators that exacerbate neuroinflammation after SD could guide targeted therapies. This review aimed to explore both the neuroinflammatory effects of SD and the impact of experimentally induced inflammatory states on SD characteristics.

**Methods:**

PubMed and Scopus were systematically searched for preclinical studies that examined the effects of SD on inflammation, and the effects of an inflammatory state on SD responses. Data extracted included authors, publication details, study type, animal characteristics, group sizes, exclusions, relevant findings, and limitations. Additional details were collected for studies on SD and neuroinflammation, including induction methods, inflammatory markers and SD characteristics in altered inflammatory states.

**Results:**

Several studies indicated that SD triggered a robust neuroinflammatory response, marked by upregulation of cytokines—interleukin-1β, tumour necrosis factor-α, and interleukin-6—alongside transcription factors such as nuclear factor kappa B, and activation of astrocytes and microglia. Key mediators including toll-like receptors, cyclooxygenase-2 and high mobility group box 1 were also implicated, with evidence of neurogenic involvement via the release of calcitonin gene-related peptide. Differences in inflammatory responses were identified between single and multiple SD induction.

Studies measuring the effect of altered inflammatory states on SD propagation were limited. Models of peripheral inflammation and non-demyelinating autoimmune encephalomyelitis did not lead to significant alterations in SD characteristics. However, administration of tumour necrosis factor was able to reduce SD amplitude, suggesting a possible neuroprotective effect.

**Conclusion:**

This review suggests potential mechanisms underlying the role of SD in neurological disorders. While SD is associated with inflammatory markers, evidence for the impact of heightened inflammatory states on cortical susceptibility to SD remains limited. Significant methodological variability and inflammatory disease models underscores the need for standardization to validate these findings. Further research into these mechanisms could identify novel therapeutic targets to mitigate SD-related neuroinflammation in neurological disorders.

**Supplementary Information:**

The online version contains supplementary material available at 10.1186/s12974-025-03503-6.

## Background

Spreading depolarization (SD) is a wave of neuronal and glial cell depolarization which slowly propagates (2–5 mm/min) across the cerebral cortex [1]. This massive depolarization triggers a loss of ion homeostasis and high resulting energy demand to correct this [2]. Typically, SD does not cause long-term cellular damage in healthy tissue and leads to a hyperaemic response to support the increased demand for respiration [2]. In pathologic tissue, however, this may instead result in ischaemia thereby exacerbating brain injury and worsening outcomes [3–5].

SD has been observed in various conditions, including stroke [6], traumatic brain injury [7], subarachnoid haemorrhage [8] and is thought to be the underlying mechanism of migraine aura [9]. Although the primary causes of these disorders differ, they share several pathological features, including heightened neuroinflammation [10–12].

SD has been shown to modify inflammatory pathways in rodents, including induction of reactive astrocytosis [13] and activation of microglia [14]. These processes may promote inflammation through release of various inflammatory mediators such as cytokines and chemokines [15]. Examples of inflammatory markers previously shown to be upregulated by SD include the pro-inflammatory cytokines interleukin-1 beta (IL-1β), tumour necrosis factor alpha (TNF-α), interleukin-6 (IL-6) and the enzyme cyclooxygenase-2 (COX-2) [16, 17]. Furthermore, neuroinflammation has been suggested to play a role in the pathogenesis of the SD wave itself, with application of proinflammatory cytokines modifying SD characteristics including amplitude and propagation velocity [18].

Identifying inflammatory mediators which exacerbate neuroinflammation following SD could inform future targeted therapies. Despite this, the precise mechanisms by which SD promotes neuroinflammation are not currently well understood. This review aims to elucidate the effects of triggering SD on neuroinflammatory pathways, as well as how alterations in inflammatory states influence the characteristics of SD, through a systematic analysis of preclinical studies—including in vitro, ex vivo, and in vivo models. This may yield new insights into the pathophysiology of SD-induced damage in neurological disorders, potentially providing targets for the development of new therapeutics.

## Methods

### Review registration

The protocol for this systematic review was prospectively registered on PROSPERO in 2024 (Protocol: CRD42024563056). The review was conducted in accordance with the Preferred Reporting Items for Systematic Reviews and Meta-Analyses (PRISMA) guidelines [19].

### Search strategy

Electronic databases PubMed and Scopus were searched in July 2024 to identify all relevant research articles using the following search terms: ((cortical spreading depression) OR (cortical spreading depolarisation) OR (cortical spreading depolarization) OR (spreading depression) OR (spreading depolarization) OR (peri-infarct depolarization)) AND ((neuroinflammation) OR (neurogenic inflammation) OR (inflammation) OR (inflammatory) OR (neuroinflammatory)).

### Inclusion and exclusion criteria

The inclusion criteria were developed based on the Population, Intervention, Comparison and Outcome (PICO) model (Supplementary Table 1). In vivo, in vitro and ex vivo pre-clinical studies were included. All methods of SD induction were included.

Two groups of studies were identified for inclusion: (1) studies which investigated the effects of SD on inflammation and (2) studies measuring the effects of altered inflammatory states on SD characteristics. Studies measuring markers which are implicated in inflammation but non-specific, such as upstream signalling molecules, were not included. Studies with human subjects and review articles were excluded, as were non-English publications.

### Study selection

Two reviewers independently conducted the search and screened all articles by title and abstract. Those which were eligible for full-text screening were then screened further according to the eligibility criteria (Supplementary Table 1). Any discrepancies between the two reviewers at any stage were resolved by a third reviewer. A total of 30 studies were included in the final review.

### Data extraction

The following information was extracted from each included study: authors, year of publication, title, study type, species and sex of animals used, total number of animals used in the experiment, total number of animals used for analysis, number of animals excluded and reason, number of animals in intervention group, number of animals in control group, additional information of relevance and study limitations.

For studies measuring the effects of SD on neuroinflammation the following additional data was extracted: description of SD induction method (in the intervention group) including dose and frequency (if applicable), description of sham procedure or vehicle in the control group, details of inflammatory markers measured and reported findings for each marker in each group.

For studies measuring the effects of altered inflammatory states on SD characteristics the following additional data was extracted: description of method of altering inflammatory state (in the intervention group), the dose and frequency (if applicable), description of sham procedure or vehicle (if used) in the control group, description of SD induction method (in both groups) including dose and frequency, details of the SD characteristics measured and reported findings for each characteristic in each group.

### Quality assessment and risk of bias

The Systematic Review Centre for Laboratory Animal Experimentation (SYRCLE) quality assessment checklist was used to assess the quality and risk of bias in the included studies [20]. Each study was screened using 10 signalling questions – a “yes” was only recorded if there was explicit reference made regarding efforts to combat the specific bias; otherwise, a “no” was recorded. This was used to generate a risk of bias score from 0–10, with 0 being the highest and 10 being the lowest risk of bias.

## Results

**Fig. 1:**
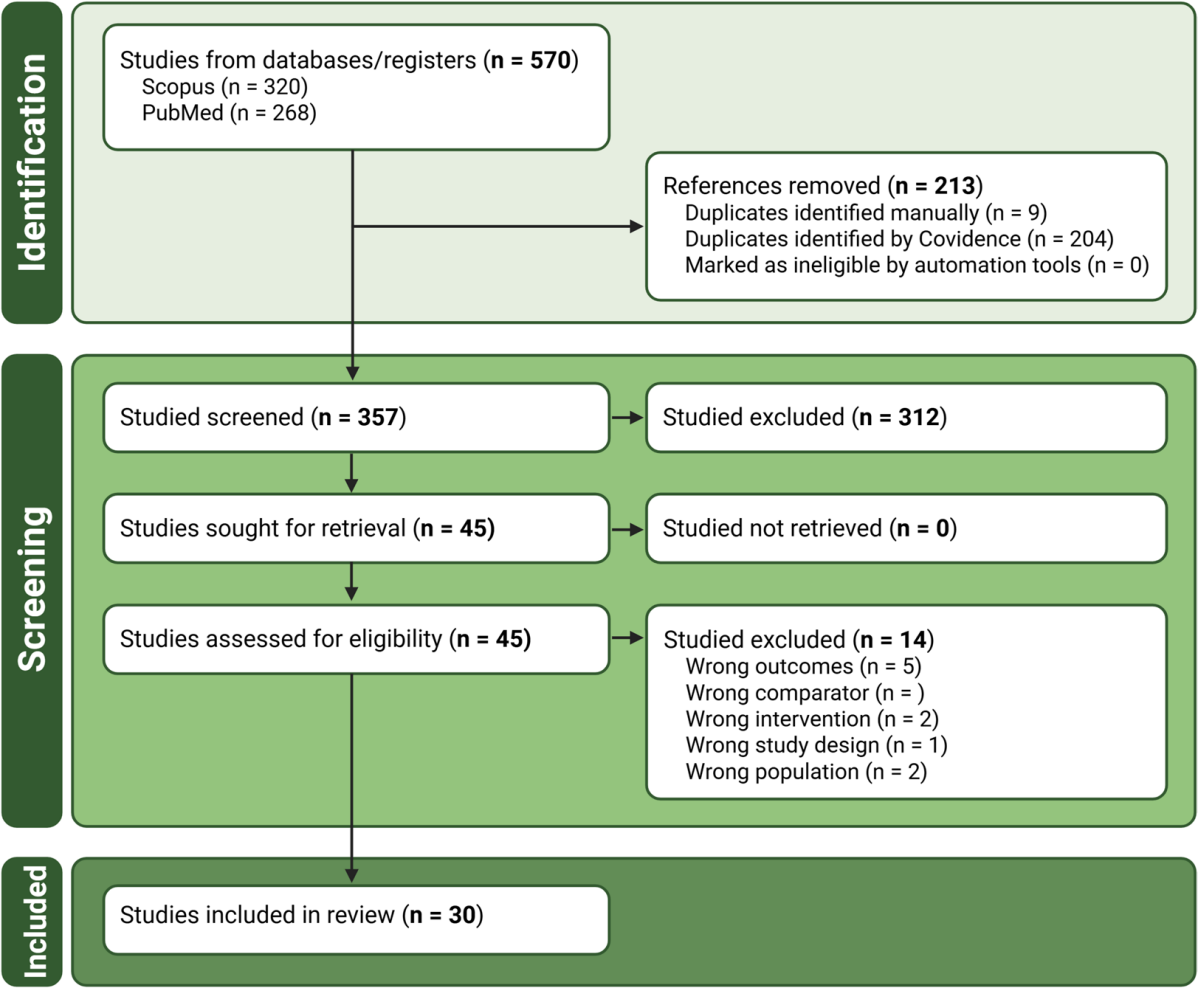
PRISMA flow diagram showing study identification, screening, and inclusion with counts (n)

Database searches identified 268 records from PubMed and 302 from Scopus, of which 213 were duplicates. This left 357 studies which were retrieved for title and abstract screening, yielding 45 studies suitable for full-text review, of which 30 were deemed eligible for inclusion in the final analyses (Fig. 1).

### Summary of methods used to induce SD

**Fig. 2:**
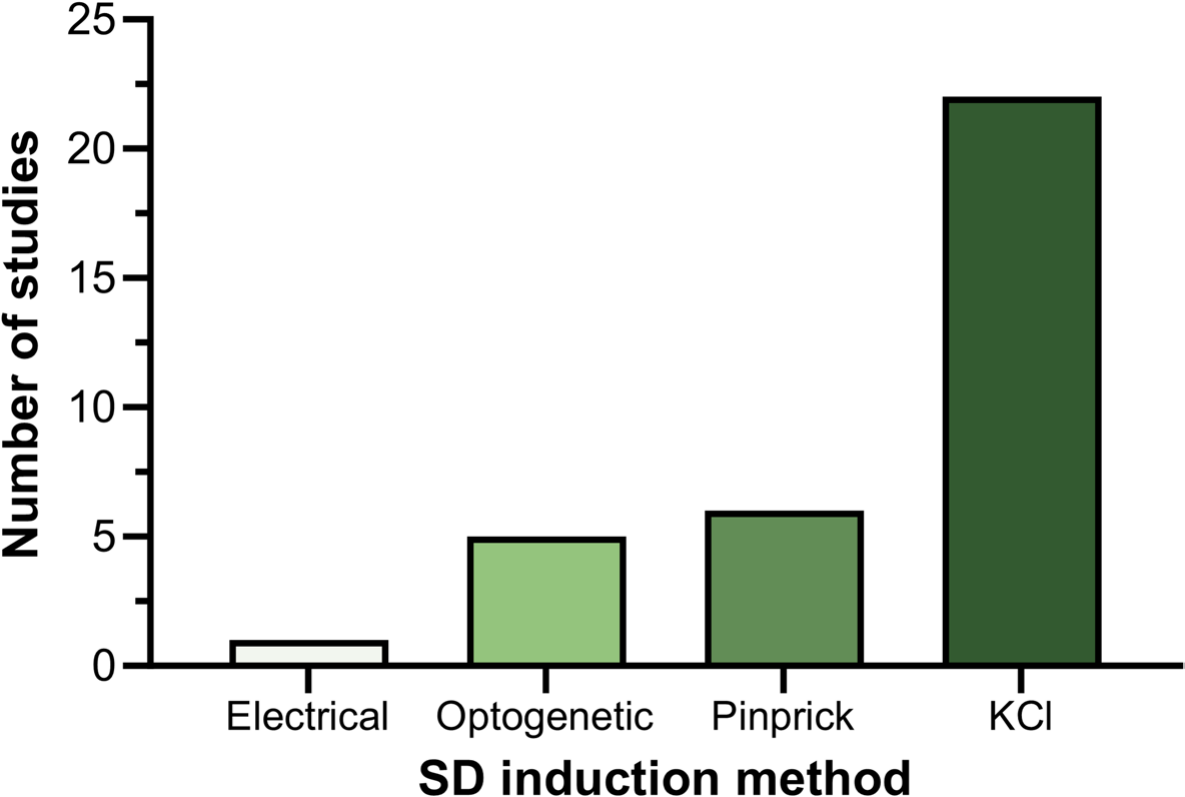
Number of studies using each method to induce spreading depolarisation (SD). Methods included electrical stimulation, optogenetic, pinprick, and potassium chloride (KCl) application

A variety of methods were used to induce SD (Fig. 2). Topical application of potassium chloride (KCl) to cortical tissue is a well-established method for inducing SD [21] and was the most commonly used across studies, with concentrations ranging from 260 mM to 3 M. Other methods used to induce SD included controlled pinprick, optogenetic light stimulation and direct electrical stimulation (Fig. 2).

### Biochemical measurements of inflammatory changes

We identified 27 studies measuring the effects of SD on neuroinflammation. The inflammatory markers which were measured included pattern recognition receptors (PRRs), cytokines and chemokines, inflammatory enzymes and transcription factors. Other readouts of inflammation included cell morphology and markers of neuroinflammatory cell activation, namely astrocytes and microglia, neuropeptide markers of neurogenic inflammation, inflammatory gene expression and measures of the physiological effects of inflammation, such as plasma extravasation or leukocyte migration.

The most studied markers were IL-1β (12 studies) [13, 16, 17, 22–30], TNF-α (10 studies) [13, 16, 22–25, 28, 30, 31], IL-6 (6 studies) [13, 16, 24, 25, 27, 28]and COX-2 (8 studies) [16, 17, 25–27, 30, 32, 33](Table 1). A full list of all inflammatory markers included across all studies can be found in Table 2.

**Table 1:** Commonly measured markers of inflammation following SD

Inflammatory marker	Upregulated (significantly or qualitatively)	Sample type	No/non-significant change	Sample type
IL-1β	[13, 16, 17, 22–25, 29, 30]	Ex vivo brain slices Cultured astrocytes Cortical tissue CSF-enriched solutions	[16, 27, 28]	Plasma Cortical tissue
TNF-α	[13, 16, 22–24, 30, 31]	Ex vivo brain slices Cultured astrocytes Cortical tissue	[16, 25, 28]	Plasma Cortical tissue
IL-6	[13, 16, 25]	Ex vivo brain slices Cultured astrocytes Cortical tissue	[16, 24, 27, 28]	Plasma Cortical tissue
COX-2	[16, 17, 25–27, 30, 32, 33]	Cortical tissue		
HMGB1	[17, 30, 34–36]	Cortical tissue		
Microglial activation (cell number, volume, morphological changes)	[14, 27, 30, 37, 38]	Cortical tissue	[36, 39]	Cortical tissue
CGRP	[24, 26, 30, 33]	Cortical tissue Trigeminal nerve		
NF-κB	[17, 25, 34]	Cortical tissue		
CCL2	[16, 30]	Cortical tissue		
GFAP	[13, 39]	Ex vivo brain slices Cultured astrocytes	[27, 38]	Ex-vivo brain slices Cortical tissue
VCAM-1	[25]	Cortical tissue	[16]	Cortical tissue
Caspase-1	[17, 30]	Cortical tissue		
IL-4			[25, 31]	Cortical tissue
Plasma extravasation			[40, 41]	Dura mater Pial microvessels

**Table 2:** Overview of studies measuring the effect of SD on inflammatory markers

Study	Model	Experimental groups	SD induction method	Single/multiple SDs	Control group conditions	Inflammatory markers measured	Effect on inflammatory markers	Time of measurement relative to first SD event (change in measurement intervention vs control)
Loonen et al*.* (2022) [77]	Female WT mice	SD: *n* = 10 animals Controls: *n* = 5 animals	KCl	Multiple	NaCl	PGD2, DPAn-3, EPA, ALA, DHA	PGD2 raised in WT mice at 0.5hand back at baseline by 1.5 h. DPAn-3, EPA, ALA, DHA were unchanged at 4 h but raised by 24 h, back at baseline by 48 h	30 m: PGD2 (**↑**) 1 h: PGD2 (=) 1.5 h: PGD2 (=) 4 h: PGD2 (=), DPAn-3 (=), EPA (=), ALA (=), DHA (=) 24 h: PGD2 (=), DPAn-3 (**↑**), EPA (**↑**), ALA (**↑**), DHA (**↑**) 48 h: PGD2 (=), DPAn-3 (=), EPA (=), ALA (=), DHA (=)
Takizawa et al*.* (2020) [16]	Male/female Thy1-ChR2 YFP mice, WT mice	Cortical tissue measurements: Optogenetic single SD: *n* = 7 KCl single SD: *n* = 16 6 SD events Optogenetic or KCl: *n* = 40 Plasma and spleen measurements: SD: *n* = 13 Controls: *n* = 13	Optogenetic, KCl	Single, Multiple	Cortical tissue measurements: Control: contralateral hemisphere Plasma and spleen measurements: Control: sham without optogenetic stimulation	IL-1β, CCL2, TNF-α, IL-6, ICAM-1, VCAM-1, COX-2	Single SD: COX-2 and IL-1β were elevated at 1 h (IL-1β markedly); CCL2 and TNF-α increased at 4 h but less than with six SDs. IL-6, ICAM-1, and VCAM-1 showed no significant changes. IL-1β rose by 10 min, COX-2 and CCL2 by 20 min. No plasma changes were detected Six SD events: COX-2 peaked at 1 h and stayed elevated for ≥ 4 h; IL-1β peaked sharply at 1 h, normalised by 2 h; CCL2 rose at 1 h, peaked at 2–4 h, and returned to baseline by 24 h; TNF-α increased at 1–2 h, peaked at 4 h, and normalised by 24 h; IL-6 and ICAM-1 showed modest increases at 1 h/12 h and 4 h/12 h respectively; VCAM-1 remained unchanged	Single SD: 5 m: IL-1β (=), CCL2 (=), IL-6 (=), COX-2 (=) 10 m: IL-1β (↑), CCL2 (=), IL-6 (=), COX-2 (=) 20 m: IL-1β (↑), CCL2 (↑), IL-6 (=), COX-2 (↑) 1 h: COX-2 (↑), IL-1β (**↑**), IL-6 (=) 4 h: COX-2 (=), IL-1β (=), CCL2 (↑), TNF-α (↑), IL-6 (=), ICAM-1 (=), VCAM-1 (=) Multiple SDs: 1 h: COX-2 (↑), IL-1β (**↑**), CCL2 (↑), TNF-α (=), IL-6 (↑), ICAM-1 (=), VCAM-1 (=) 2 h: COX-2 (↑), IL-1β (=), CCL2 (↑), TNF-α (=), IL-6 (=), ICAM-1 (=), VCAM-1 (=) 4 h: COX-2 (↑), IL-1β (=), CCL2 (↑), TNF-α (↑), IL-6 (↑), ICAM-1 (↑), VCAM-1 (=) 12 h: COX-2 (=), IL-1β (=), CCL2 (=), TNF-α (=), IL-6 (↑), ICAM-1 (↑), VCAM-1 (=) 24 h: COX-2 (=), IL-1β (=), CCL2 (↑), TNF-α (=), IL-6 (=), ICAM-1 (=), VCAM-1 (=)
Li et al*.* (2024) [29]	Male Sprague–Dawley rats	SD: *n* = 9 animals Controls: *n* = 6 animals	KCl	Multiple	Sham surgery without KCl	IL-1β	IL-1β gene and protein expression was raised following SD	Not specified: IL-1β (↑)
Ghaemi et al*.* (2017) [13]	Male Wistar rats	SD: *n* = 16 animals Controls: *n* = 16 animals	KCl	Multiple	NaCl	Astrocyte cell volume and distribution, markers of astrocyte reactivity (GFAP, vimentin, S100β, IDO), CD11β, TNF-α, IL-6, IL-1β, TLR3, TLR4	Astrocyte volume and number increased in the ipsilateral entorhinal cortex, and volume/percentage in the somatosensory cortex, no changes in the amygdala. Astrocyte reactivity markers, CD11β, TNF-α, IL-6, IL-1β, and TLR3/4 expression were elevated	Not specified: astrocyte volume/number (↑), CD11β (↑), TNF-α (↑), IL-6 (↑), IL-1β (↑), TLR3/4 (↑)
Jander et al*.* (2001) [22]	Male Wistar rats	SD: *n* = 4 animals Controls: *n* = 4 animals	KCl	Multiple	NaCl	TNF-α, iNOS, IL-1β	TNF-α and IL-1β were elevated at 4 h, declining towards baseline by 16 h; iNOS remained unchanged	4 h: TNF-α (**↑**), IL-1β (**↑**), iNOS (=) 16 h: TNF-α (=), IL-1β (=), iNOS (=)
Dehghani et al. (2021) [34]	Female WT mice	HMGB1 release SD: *n* = 5 Controls: *n* = 5 NF-κB SD: *n* = 4 Controls: *n* = 4	Pinprick, KCl	Single (pinprick), Multiple (KCl)	Pinprick controls: Naïve group: surgery but no parietal drilling ‘Drilled’ control group: surgery and parietal drilling but no pinprick KCl controls: NaCl	HMGB1, NF-κB	Neuronal HMGB1 release increased in the ipsilateral motor cortex, striatum, and contralateral striatum; astrocytic NF-κB translocation rose in both motor cortex and striatum bilaterally	Single SD: 30 m: HMGB1 (**↑**), NF-κB (**↑)** Multiple SDs: Not specified: NF-κB (-**)**
Eising et al*.* (2016) [78]	Male WT mice	SD: *n* = 6 animals Controls: *n* = 6 animals	KCl	Multiple	NaCl	Gene expression levels grouped by function	SD upregulated genes linked to interferon-mediated immunity, cytokine/chemokine signalling, and defence responses, including increased expression of Isg15, Ctsz, and other interferon-related genes	24 h: Genes in interferon-mediated immunity, cytokine/chemokine signalling and defence response groups (**↑)**
Sosthenes et al*.* (2019) [37]	Male Wistar rats	SD: *n* = 6 animals Controls: *n* = 6 animals	KCl	Multiple	Sham surgery without KCl	Iba1-positive microglia	Egr-1 + cell density was quantified, and high-density regions were stained for microglia (Iba-1). Microglial morphological changes were detected in the contralateral hemisphere of the 6 h high-SD group, but not ipsilaterally in 2 h or 6 h groups	Not specified: Microglial morphological changes (=)
Karatas et al*.* (2013) [17]	Swiss albino mice	SD: *n* = 3 animals Controls: *n* = 3 animals For each measurement	Pinprick, KCl	Single (pinprick), Multiple (KCl)	Sham surgery without KCl	Caspase-1, HMGB1, IL-1β, NF-κB, COX-2, iNOS, mast cell degranulation	Neuronal caspase-1 activation occurred within 5 min of SD, accompanied by elevated HMGB1 and IL-1β. NF-κB translocation was observed in most astrocytes by 30 min, with COX-2 and iNOS induced in astrocytes of the glia limitans. Mast cell degranulation percentage also increased	Single SD: 5 m: Caspase-1 (↑) 30 m: NF-κB (↑), mast cell degranulation (**↑**) Not specified: COX-2 (**↑**), iNOS (**↑**) Multiple SD: 5 m: HMGB1 (=) 30 m: HMGB1 (↑) Not specified: IL-1β (↑)
Nie et al*.* (2021) [23]	Male Sprague–Dawley rats	SD: *n* = 7 animals Controls: *n* = 7 animals	KCl	Single	No intervention	IL-1β, TNF-α	IL-1β and TNF-α were raised	Not specified: IL-1β (↑), TNF-α (↑)
Volobueva et al*.* (2023) [24]	Male Wistar rats	SD: *n* = 9 animals Controls: *n* = 9 animals	Pinprick	Single	Homologous areas of the contralateral sham-treated cortex	IL-1β, TNF-α, IL6, TGF-β, CGRP	At 3 h post-SD, IL-1β, TNF-α, IL-6, and TGF-β were elevated in perilesional ipsilateral tissue, while only IL-1β, TNF-α, and CGRP were increased in uninjured ipsilateral tissue versus control	3 h: IL-1β (**↑**), TNF-α (**↑**),IL-6 (=), CGRP (**↑**),TGF-β (=)
Chen et al*.* (2023) [30]):	Male Sprague–Dawley rats and C57BL/6 male mice, male/female Thy1-ChR2-YFP mice	SD: *n* = 3–7 animals depending on assay Controls: *n* = 3–7 animals depending on assay	KCl, Optogenetic	Single, Multiple	Sham surgery without KCl	NLRP1, NLRP2, NLRP3, cleaved caspase-1, ASC, IL-1β, COX-2, TNF-α, CCL2, HMGB1, GSDMD, microglial activation, CGRP	NLRP3, caspase-1, and ASC were upregulated after multiple SDs, with NLRP3 and caspase-1 also elevated after a single SD; NLRP1/2 were unchanged. NLRP3 was increased in neurons, not glia. IL-1β, COX-2, TNF-α, and CCL2 were upregulated post-multiple SDs; IL-1β release and neuronal HMGB1 and GSDMD expression increased after single or multiple SDs. TNF-α was the only inflammatory marker elevated in astrocytes post-multiple SDs. Microglial number and morphology changed over time after multiple, but not single, SDs. MMA dilated and CGRP rose in trigeminal ganglion. NLRP3 activation occurred within 15 min of SD	Single SD: 15 m: NLRP-ASC (**↑)** 30 m: NLRP-ASC (↑), microglial activation (=) 2 h: Caspase-1 (↑), TNF-α (↑), CCL2 (↑) Multiple SDs: 2 h: NLRP1 (=), NLRP2 (=), NLRP3 (**↑**), NLRP3-ASC (**↑**), Caspase-1 (**↑**), ASC (**↑**), COX-2 (**↑**), TNF-α (**↑**), CCL2 (**↑**), HMGB1 (**↑**), IL-1β (**↑**), GSDMD (**↑**), CGRP (**↑**), microglial activation (**↑**)
Liu et al*.* (2022) [33]	Male Sprague–Dawley rats	CGRP: SD: *n* = 3 animals Controls: *n* = 3 animals COX-2: SD: *n* = 6 animals Controls: *n* = 6 animals	KCl	Multiple	Sham surgery without KCl	COX-2, CGRP	COX-2 was upregulated in the cortex, but not in the trigeminal ganglion or nucleus caudalis; CGRP expression increased in the trigeminal ganglion	3 h: COX-2 (↑), CGRP (↑)
Muramatsu et al*.* (2006) [79]	Male Sprague–Dawley rats	SD: *n* = 4 animals Controls: *n* = 4 animals	KCl	Multiple	NaCl	TNF-α	TNF-α levels rapidly rose in the frontal, parietal, and occipital cortex after KCl. However, levels also increased in the frontal cortex with control NaCl intervention	2 h: TNF-α (↑) 4 h: TNF-α (↑) 24 h: TNF-α (=)
Thompson et al*.* (2005) [25]	Rats (unspecified species and gender)	RNA analysis: SD: *n* = 3 animals were pooled for analysis Controls: *n* = 3 animals were pooled for analysis	KCl	Multiple	NaCl	IL-1α, IL-1β, IL-6, IL-13, IL-2R α -chain, IL-β converting enzyme, IL-1R accessory protein, IL-1 receptor-related-protein, IL-2, IL-10, IL-12, VCAM-1, NCAM-1, NCAM-2, TNF-α, TNF-β, TNFR, TNFR type II, TNF-α converting enzyme, NOS, E-selectin, IL-4, IL-5, IL-7, COX-2, NF-kB p105 subunit	SD caused upregulation of IL-1α, Il-1β, IL-6, IL-13, IL-2 receptor α-chain, IL-β converting enzyme, IL-1 receptor accessory protein, IL-1 receptor-related protein for at least one time point following SD. IL-2, IL-10 and IL-12 were significantly downregulated both immediately and 48 h following SD. VCAM-1 expression was upregulated, NCAM-1/2 were suppressed. Macrophage inflammatory proteins were raised, B-integrin was suppressed. TNF-α was only slightly raised but TNF-β, TNFR, TNFR type II and TNF-α converting enzyme were all raised at least twofold. NOS, E-selectin, IL-4, IL-5, IL-7 were unaffected. COX-2 was elevated twofold. NF-κB p105 subunit was raised at least fivefold	48 h: IL-1α (↑), IL-1β (↑), IL-6 (↑), IL-13 (↑), IL-2R α -chain (↑), IL-β converting enzyme (↑), IL-1R accessory protein (↑), IL-1 receptor-related-protein (↑), IL-2 (↓), IL-10 (↓), IL-12 (↓), VCAM-1 (↑), NCAM-1 (↓), NCAM-2 (↓), TNF-α (-), TNF-β (↑), TNFR (↑), TNFR type II (↑), TNF-α converting enzyme (↑), NOS (=), E-selectin (=), IL-4 (=), IL-5 (=), IL-7 (=), COX-2 (↑), NF-kB p105 subunit (↑)
Dehghani et al*.* (2023) [35]	Male/female WT	SD: *n* = 5 animals Controls: *n* = 4 animals	Optogenetic	Multiple	4-mw light and comparison between ipsilateral and contralateral hemispheres	HMGB1	HMGB1 was elevated bilaterally at 30 min and remained raised at 24 h but no longer significantly. At 48 h post-CSD, levels were still above baseline in WT mice, but by 72 h, both ipsi- and contralateral HMGB1 levels dropped below 20%, similar to naïve mice	30-40 m: HMGB1 (**↑**) 5 h: HMGB1 (**↑**) 24 h: HMGB1 (=) 48 h: HMGB1 (=) 72 h: HMGB1 (=)
Kaya et al*.* (2023) [36]	Male Swiss albino mice, male/female Thy1-ChR2-YFP mice	SD: *n* = 3 animals Control: *n* = 4 animals	Optogenetic, Pinprick	Single	Not specified	HMGB1, Microglial morphological change	HMGB1 released by 15 min post-SD and unchanged by 5 h. Colocalization of astrocyte processes with HMGB1-immunopositive puncta found in 50% of astrocytes compared to 0% sham. No morphological changes in microglia after 24 h. NF-κB p65 translocated to astrocyte nuclei	15 m: HMGB1 (↑) 1 h: HMGB1 uptake in microglia (=) 5 h: HMGB1 (↑) Microglial morphological changes: 24 h (=)
Cui et al*.* (2009) [14]	Male Sprague–Dawley rats	^11^C-PK11195 SD: *n* = 11 animals Immunohistochemistry SD: *n* = 4 animals Controls: *n* = 4 animals	KCl	Multiple	NaCl	Microglial imaging (11C-PK11195 PET scan and OX-42 cell imaging)	^11^C-PK11195 binding potential was significantly elevated bilaterally 8 days post-SD, indicating microglial activation, confirmed by OX-42 + cell imaging	8d: Microglial activation (imaging) (↑)
Ebersberger et al*.* (2001) [40]	Male Wistar rats	SD: pinprick *n* = 7 animals Pinprick and KCl *n* = 10 animals Controls: *n* = 6	Pinprick, KCl	Single (pinprick), Multiple (KCl)	Sham surgery without insertion of DC electrodes	Dural plasma extravasation	Dural plasma extravasation unchanged following SD	Not specified: dural plasma extravasation (=)
Ghaemi et al*.* (2014) [31]	Male Wistar rats	SD: *n* = 8 animals Control: *n* = 8 animals	KCl	Multiple	Ringer solution	TNF-α, TGF-β1, IFN-γ, IL-4, GM-CSF	Following SD, levels of TNF-α, TGF-β1, IFN-γ, and GM-CSF were elevated in the brain, with no change in IL-4. In contrast, TNF-α, TGF-β1, IFN-γ, and IL-4 were all increased in the spleen	5w: TNF-α (↑), TGF-β1 (↑), IFN- γ (↑), IL-4 (=), GM-CSF (↑)
Varga et al*.* (2020) [38]	Male C57BL/6 J mice	SD: *n* = 3 animals Controls: *n* = 3 animals	Electrical stimulation	Multiple	Contralateral hemisphere	Microglial activation, GFAP	SD reduced microglial cell body area and the number of processes originating from the soma, without affecting overall microglial numbers in the ipsilateral hemisphere. An increase in perisomatic P2Y12R-positive process density was observed. GFAP was unchanged in ipsilateral hemisphere compared to contralateral after SD	1.5 h: Microglial activation (**↑**), number of microglia (**↑**), GFAP (=)
Liktor-Busa et al*.* (2023) [39]	Female Sprague Dawley rats	SD: *n* = 3–4 animals Controls: *n* = 3–4 animals	KCl	Single	Artificial cerebrospinal fluid (aCSF)	Astrocyte reactivity (GFAP) microglial activation (Iba1), PGE2	In the periaqueductal grey, GFAP levels increased by 90 min and remained elevated at 180 min, with PGE2 rising at 180 min. No changes were observed in Iba1 expression or microglial activation	30 m: GFAP (=), Iba1 (=), PGE2 (=) 1.5 h: GFAP (**↑**), Iba1 (=), PGE2 (=) 3 h: GFAP (**↑**), Iba1 (=), PGE2 (**↑**)
Chen et al*.* (2017) [26]	Male Sprague Dawley rats and C57BL/6 J mice	SD: *n* = 6 animals Controls: *n* = 5 animals	Pinprick	Multiple	Sham surgery with craniotomy but no SD	COX-2, CGRP	COX-2 was upregulated in the cortex but not in the cerebellum, while CGRP was increased in the trigeminal ganglion with no change in the cerebellum	4 h: COX-2 (**↑**), CGRP (**↑**)
Maneesri et al. (2004) [41]	Male Wistar rats	SD: *n* = 10 animals Controls: *n* = 10 animals	KCl	Single	NaCl	Leukocyte adhesion and migration, plasma extravasation	Leukocyte adhesion and migration were unaffected; pial microvascular dilation increased without evidence of extravasation, indicating an intact blood–brain barrier	2 h: Leukocyte adhesion and migration (=), pial microvascular (↑)
Urbach et al*.* (2006) [27]	Male Wistar rats	At each time point (3 h, 24 h, 7 days, 30 days): SD: *n* = 10 animals Controls: *n* = 6 animals	KCl	Multiple	NaCl	Gene microarray including 14 000 UniGeneclusters	Immune-related genes including CCL2, CCL3, CCL4, and COX-2 were upregulated at 3 h, with FcγRIII, IL1RAP, CD74, and LGALS3 also elevated at various timepoints. GFAP, IL-1β, and IL-6 showed < twofold increases and were deemed insignificant; MMP-9 exceeded twofold but was excluded due to low expression levels	3 h: CCL2 (↑), CCL4 (↑), COX-2 (↑), CCL3, FcγRIII (↑), CD74 (=), LGALS3 (=), IL1RAP (↑) 24 h: CCL2 (↑), CCL4 (=), COX-2 (=), CCL3 (=), FcγRIII (=), CD74 (=), LGALS3 (↑), IL1RAP (=) 7d: CCL2 (=), CCL4 (=), COX-2 (=), CCL3 (=), FcγRIII (=), CD74 (↑), LGALS3 (↑), IL1RAP (=) 30d: CCL2 (=), CCL4 (=), COX-2 (=), CCL3 (=), FcγRIII (=), CD74 (=), LGALS3 (=), IL1RAP (=) All other measured genes (including notably GFAP, IL-1β, and IL-6) were not detected at any timepoint
Miettinen et al*.* (1997) [32]	Male Wistar rats	SD: *n* = 3–5 animals Controls: *n* = 3–5 animals	KCl	Multiple	Contralateral hemisphere	COX-1, COX-2	COX-1 was undetected in all groups, while COX-2 expression increased at 4 h and 8 h post-SD, returning toward baseline by 24 h	4 h: COX-2 (↑) 8 h: COX-2 (↑) 24 h: COX-2 (=) 48 h: COX-2 (=) Not specified: COX-1 (=)
Dell’Orco et al*.* (2023) [28]	Female Thy1-ChR2 YFP and C57BL/6 J mice	KCl SD: *n* = 3 animals Controls: *n* = 3 animals Optogenetic SD: *n* = 3 animals Controls: *n* = 3 animals	KCl, Optogenetic	Multiple	NaCl (KCl group), 0.4mW light (optogenetic group)	Gene microarray	Inflammation-related genes PTGS2 and NR4A1 were upregulated at 2 h post-SD, while TNF-α, IL-1β, and IL-6 showed no change	2 h: PTGS2 (↑), NR4A1 (↑),TNF-α (=), IL-1β (=), IL-6 (=)

(↑)—increased (significantly or qualitatively), (=)—no change, (↓)—decreased (significantly or qualitatively), *D* days, *h* hours, *m* minutes

IL-1β expression levels were significantly raised in animals following SD compared to controls in 10 of the studies for ≥ one measured timepoint following SD [13, 16, 17, 22–26, 29, 30]. The two studies which did not report significant changes measured *IL-1β* expression via gene microarray. Dell’Orco, M et al. found that *IL-1β* expression was not upregulated 2 h post-SD [28], whilst Urbach, A et al. found expression was increased but not significantly (< twofold increase) [27].

TNF-α expression levels were significantly raised post-SD for at least one measured timepoint in seven of the 9 studies [13, 16, 22–24, 30, 31]. Thompson, C et al. reported increased TNF-α but did not report whether this was significant [25]. Dell’Orco, M et al. however found *TNF-α* gene expression was not significantly upregulated 2 h post-SD [28].

IL-6 levels were significantly raised following induction of a variable number of SD events for at least one measured timepoint in three studies [13, 16, 25]. Of the three remaining studies, two measured *IL-6* expression via gene microarray. In a study by Urbach, A et al., levels were raised but not significantly (< twofold) [27], while the Dell’Orco, M et al. demonstrated that *IL-6* expression was not found to be significantly upregulated two h post-SD [28]. A study by Volobueva, M et al. reported *IL-6* mRNA levels were not significantly raised following SD in the ipsilateral cortex compared to the sham-treated contralateral hemisphere when accounting for the local effects of the SD pinprick injury [24].

In seven of the eight studies measuring COX-2, significant upregulation was reported post-SD when compared to control [16, 25–27, 30, 32, 33]. In the remaining study by Karatas, H et al., induction of COX-2 in astrocytes was reported following SD but was not quantified [17].

Other inflammatory markers which were commonly measured included the nuclear protein high mobility group box 1 (HMGB1) (five studies) [17, 30, 34–36] and the transcription factor nuclear factor-kappa B (NF-κB) (three studies) [17, 25, 34]. Three studies which quantified HMGB1 levels using immunohistochemistry and western blot all reported significant increases following SD when compared to controls [17, 34, 35]. The remaining studies used immunofluorescent labelling and reported that SD induced HMGB1 release from cells [30, 36], with one of the studies also reporting SD caused colocalization of HMGB1-immunopositive puncta with astrocytes, but not microglia [36]. The three studies measuring NF-κB reported a significant increase in either NF-κB translocation in astrocytes [17, 34], or expression (specifically of the p105 subunit) following SD [25].

Calcitonin gene-related peptide (CGRP) is a vasoactive neuropeptide which is involved in the vasodilatory component of neurogenic inflammation, rather than the cytokine-driven immune-mediated inflammation [42]. CGRP was found to be upregulated in four studies following SD, as measured by miRNA expression, immunofluorescence and western blot [24, 26, 30, 33].

### Characterisation of neuroinflammatory cell responses

Several studies chose to measure the effects of SD on neuroinflammatory cells, namely astrocytes [13, 27, 38, 39] and microglia [14, 27, 30, 36–39]. Ghaemi, A et al. reported that multiple SDs significantly increased the volume and number of astrocytes, but not neurons, in the ipsilateral entorhinal and somatosensory cortices, but not the amygdala [13]. This was accompanied by a significant increase in expression of the astrocyte markers glial fibrillary acidic protein (GFAP), vimentin and S100 calcium-binding protein B (S100B) in the entorhinal cortex [13]. Liktor-Busa, E et al. also reported that a single SD increased GFAP detection in the periaqueductal grey after 90 min and this remained elevated at 180 min [39]. A further study by Urbach et al. measuring gene expression after multiple SDs found a slight increase in *GFAP* gene expression, but this was not deemed to be significant (< twofold increase) [27]. Varga et al. also did not find a significant change in GFAP expression 90 min after SD, but used the contralateral hemisphere as the control [38].

Four of the studies investigating the effects of SD on microglia determined that multiple SD events were able to induce activation of microglia [14, 27, 30, 38]. However, a study by Chen, P et al. found that these effects were not detected after only a single SD event [30]. Two additional studies found that a single SD event was insufficient to induce microglial activation [36, 39]. A study by Sosthenes, M et al. investigated regions of the brain with a high density of the transcription factor EGR-1 after SD and found microglial morphological changes in the contralateral but not ipsilateral hemisphere [37].

### The impact of neuroinflammation on SD

We identified three studies measuring the impact of neuroinflammation on SD characteristics (Table 3). These included models of peripheral inflammation [43], traumatic and ischaemic brain injury [18] and a model of acute disseminated encephalomyelitis [44].

**Table 3:** Overview of studies measuring effect of inflammation on SD characteristics

Study	Animals	Experimental groups	SD induction method	Inflammatory disease model	Control group conditions	SD characteristics measured	Effect on SD characteristics
Richter et al*.* (2014) [18]	Male Wistar rats	TNF: 0.005 ng: *n* = 7 animals 0.05 ng: *n* = 7 animals 0.5 ng: *n* = 8 animals 5 ng: *n* = 7 animals Controls: *n* = 3 animals	KCl	Administration of TNF to the cortex in a range of concentrations (0.005, 0.05, 0.5, 5 ng) to model brain injury	Administration of aCSF	Amplitude	TNF reduced SD amplitude in a dose-dependent manner, with 5 ng completely blocking SD. This effect was prevented by anti-TNFR2, but not anti-TNFR1 antibodies
Liu et al*.* (2021) [43]	Male and female mice, PC::G5-tdT, Iba1-Cre, Cx3cr1-CreER	Lipopolysaccharides: *n* = 3 animals Controls: *n* = 3 animals	KCl	Intraperitoneal injection of lipopolysaccharides 12 h prior to SD, to model peripheral inflammation)	Intraperitoneal injection of saline	Velocity	Wavefront velocity was unchanged compared to controls
Merkler et al*.* (2009) [44]	Female Lewis rats and C57Bl/6 mice	Immunised: *n* = 9 animals Controls: *n* = 9 animals	KCl	TNF-α and IFN-γ injected into animals previously immunised against guinea pig MBP72-85 (model of non-demyelinating acute disseminated encephalomyelitis)	Contralateral hemisphere	Velocity	SD velocity was unaltered by the autoimmune encephalomyelitis model after 3 and 8 days

Administration of lipopolysaccharide to model peripheral inflammation in mice prior to SD induction did not significantly alter the wavefront velocity [43]. In a generalised model of brain injury and ischaemia, pre-treatment with TNF before SD induction led to a dose-dependent reduction in SD amplitude, with the highest dose (5 ng) completely blocking SD [18].

A study by Merkler, D et al. modelled autoimmune demyelinating diseases by immunizing mice with recombinant myelin oligodendrocyte glycoprotein (rMOG) and sensitized them using a cytokine mixture of TNF-α and IFN-γ [44]. This approach induced widespread subpial demyelination and cortical infiltration of macrophages and microglia. SD propagation was significantly accelerated in the treatment group. In the same study, mice were also immunized with guinea pig myelin basic protein peptide 72–85 (MBP72–85) to model inflammation without demyelination, resulting in perivascular T-cell and macrophage infiltration and blood–brain barrier breakdown, resembling acute disseminated encephalomyelitis but without demyelination [44]. In this model, no significant difference in SD velocity was found between the injected hemisphere and control contralateral hemisphere [44].

### Quality appraisal

**Fig. 3:**
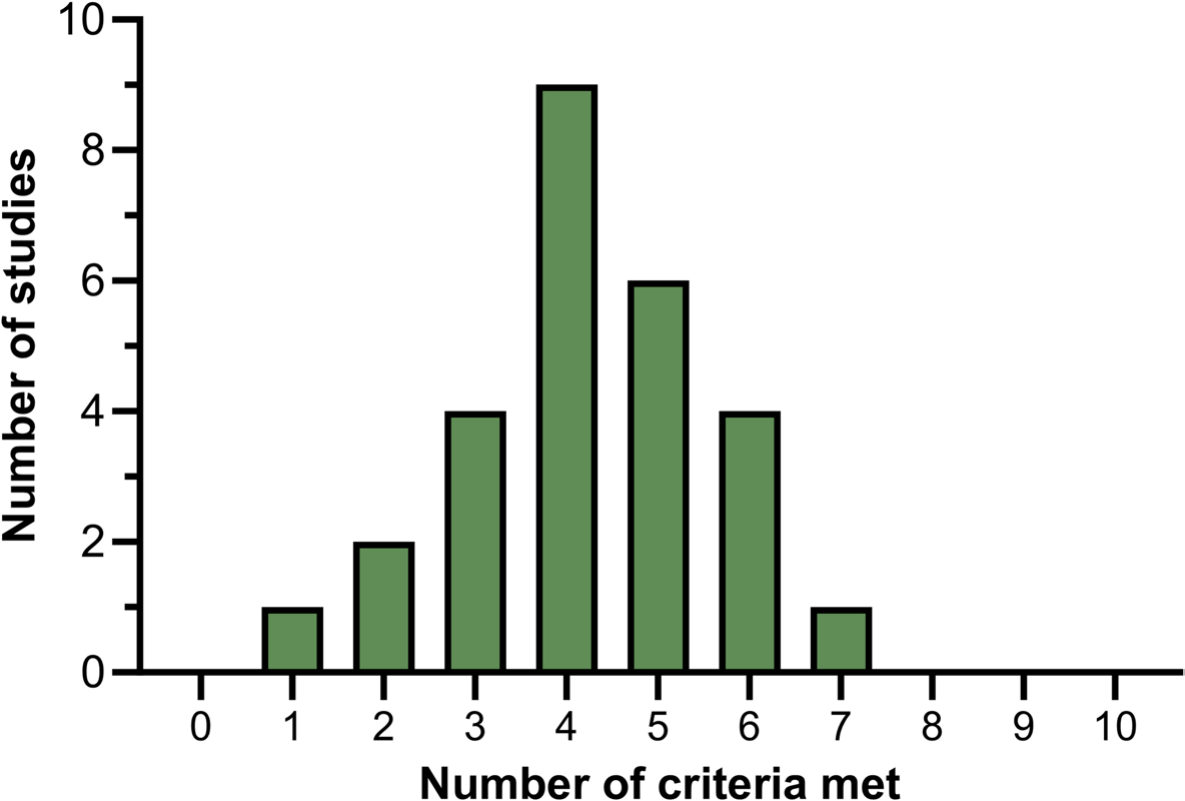
Number of studies meeting SYRCLE risk of bias (RoB) assessment criteria

The SYRCLE Risk of Bias (RoB) tool was used to assess the risk of bias of the included studies (Table 4). Compliance across the included studies was generally low (Fig. 3). The domains of selection bias, performance bias and detection bias were the least compliant, with very few studies outlining robust attempts made to blind investigators to the sequence generation, allocation, intervention and assessment phases. However, most studies were found to be free of the effects of attrition bias and selective outcome reporting.

**Table 4:**
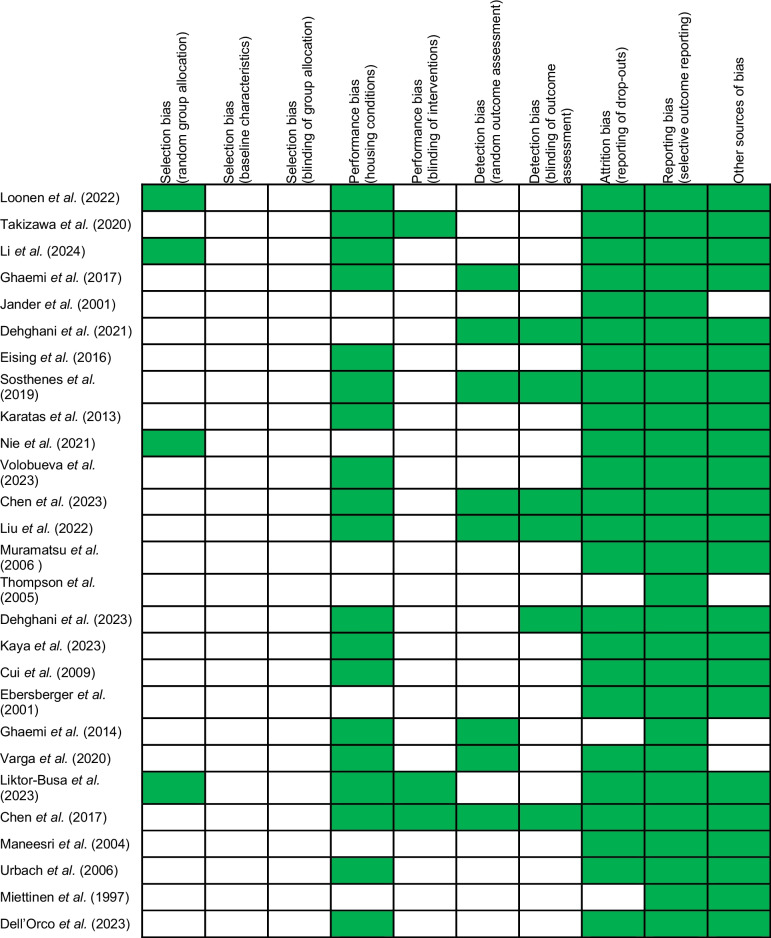
SYRCLE Risk of Bias tool. Green – No bias. White – signs of bias or not specified [13, 14, 16, 17, 22–38, 40, 41, 77–79]

## Discussion

SD is implicated in several neurological and neurovascular disorders [6–9], which are often characterised by neuroinflammation [10–12]. Investigating a potential bidirectional relationship between SD and inflammation may yield new therapeutic targets for neurological disorders which are worsened by SD. We therefore set out to investigate if SD induces neuroinflammatory effects and if increased inflammatory states results in exacerbated SD responses.

**Fig. 4:**
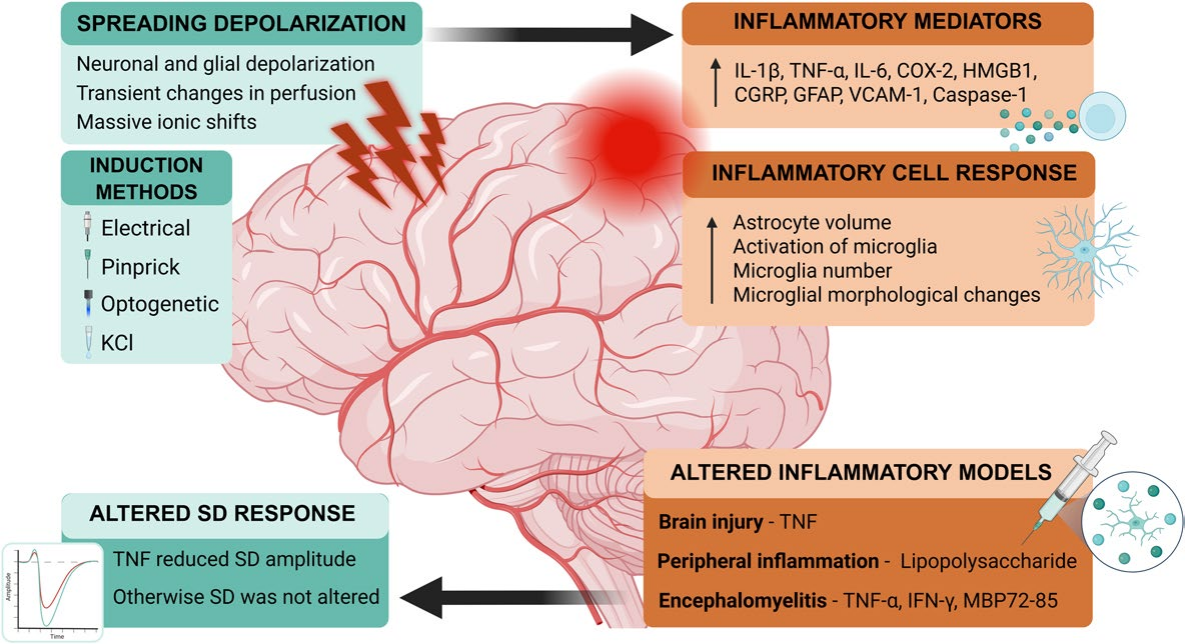
Overview of the results of the systematic review. Spreading depolarization (SD), induced via electrical, pinprick, optogenetic, or potassium chloride (KCl) stimulation, triggered upregulation of inflammatory mediators and cellular responses. In models of altered inflammation, including brain injury, peripheral inflammation, and encephalomyelitis, only TNF administration reduced SD amplitude

The majority of studies found an upregulation in inflammatory markers following SD, including IL-1β [13, 16, 17, 22–26, 29, 30], TNF-α [13, 16, 22–24, 30, 31], IL-6 [13, 16, 25] and COX-2 [16, 17, 25–27, 30, 32, 33] (Fig. 4). This was accompanied by evidence of neuroinflammatory cell activation, including reactive astrogliosis and microglial morphological changes [14, 27, 30, 37, 38]. Several studies also reported an increase in extracellular HMGB1 release [17, 30, 34–36] and NF-κB signalling [17, 25, 34], indicating a sustained neuroinflammatory response. Additionally, upregulation of CGRP [24, 26, 30, 33] was also reported, suggesting a neurogenic inflammatory component.

Three studies examined the effects of altered inflammatory states on SD characteristics, yielding mixed evidence, likely due to the diversity of models used [18, 43, 44], with one study reporting a reduction in SD amplitude following inflammation [18].

### The effect of methodological variability in SD induction on inflammatory profiles

There was wide variation in the methods used to induce SD. While most studies used KCl to induce SD, there were several studies which induced SD via pinprick for at least one subgroup [17, 24, 26, 34, 36, 40]. SD induction often involves craniotomy, which has been shown to confound the inflammatory response [45]. Therefore, cortical injuries, caused by either pinprick or invasive procedures, may have influenced study findings. Three studies comparing optogenetic-induced SD with invasive methods found no differences in inflammatory markers, suggesting that the method of SD induction did not influence the outcomes [16, 30, 36].

### SD elicits a sustained neuroinflammatory response

Inflammatory markers were assessed in various samples, including ex vivo brain slices [13, 23], cultured astrocytes [13], cortical tissue [14, 16, 17, 22–39], CSF-enriched solutions [30], trigeminal ganglion [30], dura mater [40], and pial microvessels [41]. Interestingly, while significant changes in inflammatory markers were observed in cortical tissue compared to sham samples, no differences were detected in plasma. This suggests that the inflammatory changes are localized specifically to the neuronal tissue [46].

IL-1β was the most studied inflammatory cytokine in this review and was mostly found to be upregulated following SD [13, 16, 17, 22–26, 29, 30]. In a study by Takizawa, T et al., IL-1β expression was significantly increased as soon as 10 min following SD and peaked at 1 h, supporting the notion that IL-1β is one of the first inflammatory cytokines released following SD [16]. A further study suggested a possible mechanism for this increase, determining that the increase in IL-1β release following SD was dependent on the release of the NLR family pyrin domain containing 3 (NLRP3) inflammasome [30]. Knockout of the IL-1β-encoding gene in mice also ameliorated the increase in COX-2 post-SD [30]. This may suggest IL-1β may partially mediate downstream signalling following SD, with NLRP3 as the upstream mediator of this inflammatory axis.

Other cytokines which were commonly found to be raised following SD in the cortical tissue, include TNF-α [13, 16, 22–24, 30, 31] and IL-6 [13, 16, 25]. Knockout of the gene encoding interleukin-1 receptor (IL-1R) gene resulted in a blunting of the TNF-α response following SD in one study [16]. Another study found that administration of TLR2/4 was able to suppress microglial activation following SD without reducing IL-1β levels yet was still able to cause a reduction in cortical TNF-α mRNA expression [30]. These findings may suggest that the rise in TNF-α following SD occurs due to a combination of upstream IL-1β action and microglial modulation. IL-6 exhibits both pro- and anti-inflammatory properties and contributes to the induction of acute-phase responses [47]. It plays a key anti-inflammatory role in local and systemic acute inflammation by modulating proinflammatory cytokine levels without affecting anti-inflammatory cytokines [47]. The mixed findings following SD [13, 16, 24, 25, 27, 28] may reflect the timing of measurement, as IL-6 may exhibit a biphasic response following stimulation.

HMGB1 is a nuclear protein with multiple functions including action as a damage-associated molecular pattern (DAMP) molecule [48]. All five studies measuring HMGB1 post-SD reported an increase in either HMGB1 expression or release [17, 30, 34–36]. One study also reported colocalization of HMGB1-immunopositive puncta with astrocytes and microglia and found that HMGB1 was taken up by perisomatic astrocyte processes, but not microglia [36]. HMGB1 release has been associated with NF-κB p65 nuclear translocation in astrocytes [17, 34]. NF-κB is a nuclear transcription factor which can induce the expression of pro-inflammatory genes and may also be involved in regulation of the inflammasome [49]. NF-κB activation in astrocytes has also been suggested to play a role in COX-2 increase observed following SD [17]. Therefore, it is likely that HMGB1 acts on astrocytes to promote the upregulation of NF-κB, which may further contribute to the proliferation of the inflammatory cascade.

A range of studies explored the involvement of the neuroinflammatory cells astrocytes and microglia following SD [14, 27, 30, 37, 38]. Astrocytes undergo morphological and proliferative changes during reactive astrogliosis in response to insults to brain tissue [50], including hypertrophy, enlarged cell bodies and elongated major processes with increased thickness [51]. Additionally, microglia are highly mobile immune cells which produce proinflammatory cytokines [52, 53].

Several studies noted a rise in astrocyte-related markers such as GFAP in various areas of the brain post-SD, suggesting that SD induces reactive astrocytosis [13, 27, 39]. However, in one of these studies the rise was non-significant [27], and a further study did not find a significant change in GFAP levels post-SD [38]. Ghaemi et al. also found an increase in astrocyte numbers following SD, suggesting SD can cause proliferation of astrocytes in addition to activation [13], however this has not yet been replicated and requires future investigation. The study by Ghaemi et al. also found an upregulation in the expression of the pattern recognition receptors toll-like receptor (TLR) 3 and TLR4 in astrocytes [13]. Administration of poly I:C, a TLR3 ligand that suppresses proinflammatory cytokines and promotes anti-inflammatory cytokines [54], was able to significantly reduce the mean volume of astrocytes following SD [13]. This suggests that the reactive astrocytosis following SD may be in part mediated by TLR3 and TLR4. Another study found that poly I:C was able to suppress the production of TNF-α and interferon-gamma (IFN-γ) in the brain following SD, further supporting the role of TLR3 in SD-related inflammation [31]. TLR4 can promote the production of IL-6 when activated by specific ligands, and the IL-6/STAT3 axis is a known mechanism for astrogliosis [55]. Ghaemi et al. reported significantly higher levels of IL-6 in SD derived astrocytes compared to astrocytes from control animals, suggesting a possible mechanism for the SD-related astrocyte activation [13].

Microglial activation was also shown to be promoted after induction of multiple SDs [14, 27, 30, 38]. One study found that suppression of SD-triggered microglial activation by minocycline, a known inhibitor of microglia, was able to downregulate NLRP3, IL-1β and COX-2 positive cells [30]. Furthermore, inhibition of microglial TLR2/4 was able to suppress SD-related microglial activation, neuronal COX2 expression and cortical expression of TNF-α [30].

CGRP was shown to be raised following SD in all relevant studies in this review [24, 26, 30, 33], as measured by miRNA quantification [24], western blot [26], immunofluorescent staining [30, 33]. CGRP contributes to a neurogenic inflammation feedback loop, where sensory neuron activation triggers rapid release of CGRP, substance P, and prostanoids, leading to plasma extravasation and oedema, faster than typical immune cell infiltration [42, 56, 57]. CGRP expression was reduced following Nlrp3 and Il1b knockout, indicating IL-1β release via NLRP3 activation is crucial for CGRP release after SD [30]. IL-1β also promotes CGRP release through COX-2 activation [58, 59], while CGRP further amplifies neuroinflammation by enhancing IL-1β's effects on glial cells [60, 61]. Thus, CGRP is both driven by and contributes to the NLRP3–IL-1β inflammatory cascade.

### Neuroinflammation may begin to explain SD-related neurovascular dysfunction

The health of tissue has been known to influence the recovery from SD, with healthy tissue typically recovering and injured tissue having a higher likelihood of becoming ischaemic [2, 62]. This can result in exacerbation of injury, expansion of acute neuronal damage and worsening of outcomes in the injured brain [3–5, 63]. One of the ways in which SD can cause permanent ischaemic damage is through disruption of neurovascular coupling [2]. In normal tissue, the massive energy requirement needed to repolarise the cell following SD is adjusted for by vasodilation, causing an increase in cerebral blood flow (CBF) also known as a hyperaemic response [2, 64]. However, in injured or metabolically depleted tissue, an inverse response can instead be observed, with an initial vasoconstriction and reduction in CBF during depolarisation, causing spreading ischaemia and a high risk of permanent tissue damage [2, 64].

Astrocytes are key cells involved in regulating integrity of the blood–brain-barrier (BBB) as part of the neurovascular unit and are responsible for releasing vasoactive substances such as prostaglandin E₂ in response to brain hypoperfusion [65]. Moreover, proinflammatory cytokines can also alter BBB permeability [66] and microglia have been linked to modulation of CBF [67]. However, while neuroinflammation is implicated in regulation of the neurovascular unit, the exact mechanisms by which the SD-specific inflammatory cascade can cause neurovascular disruption are yet to be discerned and therefore an area for future research. In addition, future experiments should take into account the context-dependent SD effects in healthy and injured tissue [2], and attempt to ascertain if the inflammatory pathways described remain unchanged across varying degrees of tissue injury.

### Inflammatory modulation does not consistently alter spreading depolarization responses

Although the mechanism of SD initiation is complex, it is believed to involve an initial trigger, such as neuronal injury or metabolic stress, followed by glutamate release and activation of NMDA receptors [2, 21, 68]. This highlights several potential pathways through which inflammation could influence the processes underlying SD wave initiation. Inflammatory cytokines have been shown to modulate neuronal excitability through altering ion channels and promoting the release of neuroactive molecules (such as glutamate) [69, 70]. This enhancement of excitability secondary to TNF was enough to cause bursting activity, SD and seizure in rat hippocampal slice culture [71]. Furthermore, an increase in permeability of the BBB, as commonly occurs in inflammatory responses [66], has been linked to SD induction through impaired potassium homeostasis [72]. We therefore set out to assess whether increased inflammatory states were linked to altered SD characteristics, and if this could be used as a mechanism to explain the worse outcomes seen following SD in patients with neurovascular disorders [3–5].

Only three studies were identified which measured the effects of increased inflammatory states on SD characteristics. A lipopolysaccharide model of peripheral inflammation was not found to increase SD wavefront velocity [43]. An inflammatory non-demyelinating model of autoimmune encephalomyelitis also did not influence SD velocity [44]. However, this study used contralateral hemisphere as control and found that inflammation, initially confined to ipsilateral hemisphere at three days post-SD, spread to the contralateral hemisphere by day eight, possibly limiting its viability as a control. A further study found that administration of TNF in a generalised model of brain injury had a reduction in SD amplitude, possibly suggesting that TNF, particularly via its receptor TNFR2, has a neuroprotective effect to limit SD during injured states [18].

Taken together, these studies do not appear to support our hypothesis that increased inflammation predisposes to altered SD responses. On the contrary, emerging evidence suggests that microglia play a protective role by actively suppressing neuronal activity [73, 74], In response to neuronal activation, microglia sense extracellular ATP and initiate a negative feedback mechanism that suppresses neuronal firing via A1 receptor activation [73, 74]. This process helps modulate excitability and prevent excessive neuronal activation. Furthermore, microglia have been shown to limit various seizure types, reinforcing the idea that their activation does not necessarily exacerbate excitability or pathological responses [73, 74].

A key consideration in understanding SD within neurovascular disorders is the role of vasoactive mediators such as CGRP, prostanoids, and substance P. Our findings demonstrate significant upregulation of CGRP following SD [24, 26, 30, 33], highlighting its central role in the vasodilatory component of neurogenic inflammation. Additionally, significant increases in COX-2 expression were observed following SD compared to controls [16, 25–27, 30, 32, 33]. Prostanoids, produced downstream of COX-2, along with substance P, further contribute to vascular regulation and increased plasma extravasation during inflammatory responses [75]. These molecules are often already elevated in chronic inflammatory states, and since SD triggers their release, this overlap suggests that baseline inflammation may not fundamentally modify SD characteristics. Instead, SD and inflammation share common molecular pathways, supporting the conclusion that inflammatory states do not inherently alter SD responses but rather reflect an intrinsic neurovascular regulatory mechanism.

However, given the small number and variability in methodologies between studies more research, measuring a variety of SD parameters such as velocity, amplitude and threshold, in a range of inflammatory disease models is required.

### Limitations

Firstly, there were methodological differences between studies, with some administering a single SD, while others measured the effects of multiple SDs or both. Therefore, these studies should be compared with caution, as translating the findings to a clinical setting should take the number of SDs into account. There was also variation in the control groups. While most studies used a separate group of control mice in which the cortex was not stimulated, a small number of studies used the contralateral hemisphere as a control [16, 24, 32, 38, 44]. This was justified by the authors as it has previously been shown that the SD wave is confined to one hemisphere [76]. However, several of the studies which measured inflammation in both hemispheres reported changes in the contralateral hemisphere also, including HMGB1 release, NF-κB activation and morphological changes in microglia [34, 37]. Therefore, it cannot be ruled out that the inflammatory responses affect both hemispheres. Standardised methodology using clearly defined study populations, separate control groups and ideally non-invasive methods of SD induction should be implemented to assess the effect of SD on exacerbation of neuroinflammation. Only three studies met the inclusion criteria to investigate the effect of increased inflammatory states on SD characteristics [18, 43, 44]. More studies across a range of disease models are necessary before concluding whether inflammation can alter SD responses in the context of neurovascular disorders.

## Conclusions

SD is a key phenomenon in several neurological disorders which feature inflammation and is associated with worse clinical outcomes. This systematic review identified that SD elicits a sustained inflammatory response, indicated by upregulation of the inflammatory markers IL-1β, TNF-α, IL-6, COX-2, HMGB1 and NF-κB, activation of astrocytes and microglia, and promotion of neurogenic inflammatory responses, as evidenced by upregulation of CGRP. This provides evidence of the inflammatory pathways which are upregulated following SD and may provide biological insights into the pathology of these neurological disorders and how SD can worsen physiology. There is not currently enough evidence to suggest that increased inflammatory states alter SD responses. Further research using inflammatory disease models to assess the effect on SD induction may yield therapeutic targets to deter the susceptibility to further SD events. This may then provide a basis for research into the mechanisms by which SD is implicated in neurovascular disorders.

## Supplementary Information

Supplementary Material 1

## Data Availability

No datasets were generated or analysed during the current study.

## Abbreviations

BBB: Blood–Brain Barrier
CGRP: Calcitonin Gene-Related Peptide
COX-2: Cyclooxygenase-2
CSF: Cerebrospinal Fluid
DAMP: Damage-Associated Molecular Pattern
GFAP: Glial Fibrillary Acidic Protein
HMGB1: High Mobility Group Box 1
IFN-γ: Interferon-gamma
IL-1β: Interleukin-1 beta
IL-1R: Interleukin-1 Receptor
IL-6: Interleukin-6
KCl: Potassium Chloride
MBP72–85: Myelin Basic Protein peptide 72–85
miRNA: MicroRNA
NF-κB: Nuclear Factor kappa-light-chain-enhancer of Activated B cells
NLRP3: NLR family pyrin domain containing 3
PRRs: Pattern Recognition Receptors
RoB: Risk of Bias
rMOG: Recombinant Myelin Oligodendrocyte Glycoprotein
S100B: S100 Calcium-Binding Protein B
SD: Spreading Depolarization
SYRCLE: Systematic Review Centre for Laboratory Animal Experimentation
TLR: Toll-Like Receptor
TNF-α: Tumour Necrosis Factor-alpha
