# Supplementary material for: A systematic review of the causes and consequences of spreading depolarization in neuroinflammation; implications for neurovascular disorders
Source: J Neuroinflammation. 2025 Jul 9;22:178. doi: 10.1186/s12974-025-03503-6 (PMC12243393; doi:10.1186/s12974-025-03503-6)
Supplement: Supplementary file 1 — Supplementary Material 1 [file 12974_2025_3503_MOESM1_ESM.docx]

**A systematic review of the causes and consequences of cortical spreading depolarization in neuroinflammation; implications for neurovascular disorders.**

Faheem Anwar*^1^, Olivia Grech,*^1^ Caroline W Mugo,^1,2^ James A Roberts,^2^ Jessica C Hubbard,^2^ Chloe N Thomas,^2^ Alexandra J Sinclair,^1,3,4α^ Lisa J Hill.^2,4α^

**Affiliations**:

1. Metabolism and Systems Science, College of Medicine and Health, University of Birmingham, Birmingham, United Kingdom, B15 2TT
2. Department of Biomedical Sciences, School of Infection, Inflammation and Immunology, College of Medicine and Health, University of Birmingham, Birmingham, United Kingdom, B15 2TT
3. Department of Neurology, Queen Elizabeth Hospital, University Hospitals Birmingham NHS Trust, Birmingham, United Kingdom, B15 2GW
4. National Institute for Health and Care Research, Birmingham Biomedical Research Centre, University Hospitals Birmingham, Birmingham, United Kingdom, B15 2TH

**Corresponding author:**

Dr Lisa J Hill

l.j.hill@bham.ac.uk

Department of Biomedical Sciences, School of Infection, Inflammation and Immunology, College of Medicine and Health, University of Birmingham, Birmingham, United Kingdom

* These authors contributed equally and are joint first authors

α These authors contributed equally and are joint senior authors

Supplementary Table 1: Inclusion criteria

|  | **Effects of SD on inflammatory markers** | **Effects of altered inflammatory states on SD characteristics** |
| --- | --- | --- |
| **Population** | Animal studies including mice and rats | Animal studies including mice and rats |
| **Intervention** | Induction of SD (all methods, including stimulation via needle-prick, potassium chloride, inflammatory soup application, electrical, mechanical) | Disease models of altered inflammatory states (such as genetic predisposition, exposure to inflammatory agents) |
| **Comparison** | No induction of SD (sham procedure, vehicle treated e.g. NaCl instead of KCl, no intervention or contralateral hemisphere) | No altered inflammatory state (vehicle treated, no intervention or contralateral hemisphere) |
| **Outcome** | Measurement of inflammatory markers specific to either immune-mediated (such as cytokines, transcription factors, enzymes, cell-surface molecules) or neurogenic responses (e.g. CGRP, substance P), markers of neuroinflammatory cell (astrocytes, microglia) activation, downstream inflammatory responses (plasma extravasation, leukocyte migration) | Changes in SD wave characteristics (such as threshold, amplitude, velocity, frequency) |
